# Preoperative Diagnostic Accuracy and Clinicopathological Characteristics of Pilomatricoma: A Single-Institution Study of 51 Patients

**DOI:** 10.7759/cureus.83960

**Published:** 2025-05-12

**Authors:** Yu Matsui, Shohei Kitayama, Teruhiko Makino, Tadamichi Shimizu

**Affiliations:** 1 Department of Dermatology, University of Toyama, Toyama, JPN

**Keywords:** clinical diagnosis, diagnostic accuracy, histopathology (hp), pilomatricoma, retrospective study

## Abstract

Pilomatricoma is a benign adnexal tumor derived from hair matrix cells and commonly affects pediatric populations. Despite its relatively characteristic clinical presentation, presurgical diagnostic accuracy remains low in many reports. This retrospective study analyzed 55 lesions from 51 Japanese patients with histopathologically confirmed pilomatricoma treated at Toyama University Hospital between 2006 and 2024. Clinical information, including age, sex, lesion location and characteristics, and the initial clinical diagnosis, was extracted from medical records. Diagnostic accuracy was defined as concordance between the clinical diagnosis and the final pathological diagnosis. Univariate and multivariate analyses were performed to examine factors associated with a correct initial diagnosis. A Spearman’s correlation analysis was also conducted to evaluate the relationship between the duration from symptom awareness to surgical excision and the basophilic-to-shadow cell (B/S) ratio.

The overall diagnostic accuracy was 21 out of 55 cases (38.2%). Patients in the correctly diagnosed group (n = 21) were significantly younger than those in the incorrectly diagnosed group (n = 34) (median: 17 vs. 36 years, p = 0.002) and had a longer interval from symptom awareness to the initial visit (median: 300 vs. 113.5 days, p = 0.03). Bluish coloration was significantly associated with correct diagnosis (14/21, 66.7%; p = 0.005), while reddish coloration was more commonly observed among misdiagnosed cases (18/34, 52.9%; p = 0.04). The multivariate analysis did not identify any independent predictors of diagnostic accuracy. A moderate negative correlation was observed between the B/S ratio and time to surgery (ρ = -0.40, p = 0.002), consistent with the histological progression of the tumor over time. These findings underscore the complexity of clinical diagnosis and highlight the importance of routinely considering pilomatricoma in the differential diagnosis of cutaneous nodules, regardless of patient age or other clinical features. Further studies are warranted to improve early recognition of this frequently misdiagnosed tumor.

## Introduction

Pilomatricoma is a benign dermal or subcutaneous adnexal tumor derived from immature hair matrix cells, originally described by Malherbe and Chenantais in 1880 [1]. The term “pilomatrixoma” was later introduced by Forbis and Helwig in 1961 [2]. It is also known as “calcifying epithelioma of Malherbe” [3].

The disease is characterized by a bimodal age distribution, with peaks during early life (0-20 years) and middle age (50-65 years [4]. The average age of onset is approximately 4.5 years, and nearly 90% of cases occur in children under 10 years of age [5]. Clinically, pilomatricoma often presents as a firm, stony-hard nodule, typically measuring 0.3-3 cm, located on the face, neck, or upper trunk in pediatric and young adult populations [6-8].

Diagnosis can often be made clinically, supported by imaging modalities such as ultrasonography or radiography [9,10]. However, due to its variable appearance-including blister-like morphology or color changes ranging from yellowish-white to reddish-accurate presurgical identification can be challenging. Although pilomatricoma is a common benign tumor encountered in routine dermatological practice, its diagnostic accuracy at the initial visit has been reported to be surprisingly low in many studies, with values often falling below 50% despite the lesion's benign nature and relative familiarity among clinicians [11,12]. This may be partly due to its diverse clinical presentation and reporting bias, and only a limited number of case series have addressed this issue in depth.

In this study, we retrospectively analyzed 55 lesions from 51 patients with histopathologically confirmed pilomatricoma treated at our institution, aiming to clarify the clinical and histopathological characteristics and to evaluate the diagnostic accuracy at the time of initial presentation.

## Materials and methods

Study design

Patients who presented with cutaneous nodules and subsequently underwent excisional biopsy at Toyama University Hospital between January 2006 and September 2024 were retrospectively reviewed. Only cases that were histopathologically diagnosed as pilomatricoma were included in the study. A total of 55 lesions from 51 patients were analyzed. Three patients had more than one lesion resected during the study period, and all variables were assessed on a per-lesion basis. Clinical data were extracted from electronic medical records and included age, sex, lesion location, clinical diagnosis at the initial visit (made by board-certified dermatologists), lesion color, presence of telangiectasia, mobility, lesion size (maximum diameter), lesion type (subcutaneous nodule, nodule, or papule), presence or absence of muscular dystrophy as comorbidity, the duration from patient awareness of the lesion to the initial visit, and the duration from awareness to surgical excision. Lesion color was independently assessed by two board-certified dermatologists. In cases of disagreement, a consensus was reached through discussion.

Pathological data were reviewed from histological slides. The presence or absence of calcification within the tumor was recorded. In addition, the proportions of basophilic cells and shadow cells (including transitional cells) within the tumor were estimated. Areas of each cell type were manually measured on representative histological images using ImageJ version 1.37 software (National Institutes of Health, Bethesda, MD, USA). For each case, two representative histological sections taken from the central region of the tumor were selected. The basophilic-to-shadow cell (B/S) ratio was calculated by including transitional cells with condensed or crescent-shaped nuclei as part of the shadow cell population. The final B/S ratio for each lesion was determined as the average of the two measurements. Since the final diagnosis was confirmed by histopathological evaluation, the concordance between the clinical and pathological diagnoses at the initial visit was used to define the diagnostic accuracy of the presurgical assessment.

Statistical analysis

Continuous variables were summarized as medians and ranges. Univariate analyses were performed using Fisher’s exact test for categorical variables and the Mann-Whitney U test for continuous variables. Multivariate analysis was conducted using logistic regression to evaluate factors associated with correct preoperative diagnosis.

In addition, Spearman’s rank correlation coefficient was used to assess the relationship between the duration from lesion awareness to surgical excision and the B/S ratio.

A two-sided P value < 0.05 was considered statistically significant. All data were analyzed using EZR (Easy R) version 1.53 (Saitama Medical Center, Jichi Medical University, Saitama, Japan) [13].

Ethical review

The Toyama University Hospital Ethics Board approved the study and waived patient consent in line with the Declaration of Helsinki (study number: R2024209; IRB approval date: February 28, 2025).

## Results

Patient characteristics

Table 1 summarizes the clinical characteristics of 55 patients, including 25 male subjects (45.5%) and 30 female subjects (54.5%), with a median age of 26 years (range: 0-78). The median time from symptom awareness to initial consultation and surgery was 183 and 213 days, respectively. Lesions were most commonly located on the upper limbs (24 cases, 43.6%) and head (21 cases, 38.2%). Most (41 cases, 74.6%) were subcutaneous nodules. Patients were divided into correctly diagnosed (n = 21, 38.2%) and incorrectly diagnosed (n = 34, 61.8%) groups. The correctly diagnosed group tended to be younger (median: 17 vs. 36 years) and had a longer duration from awareness to the first visit (median: 300 vs. 113.5 days). Mobility and telangiectasia were observed in 30 (54.5%) and 8 (14.5%) of patients, respectively. One of the patients also had a history of muscular dystrophy, although this condition was not separately tabulated in Table 1.

**Table 1:** Basic clinical and pathological characteristics of patients with pilomatricoma. Values are presented as a number (percentage) or median (range). Patients were divided into correctly and incorrectly diagnosed groups based on the accuracy of the initial clinical diagnosis. Statistical significance was defined as p < 0.05 throughout the study.

Basic characteristics		Cases (n = 55)	Correctly diagnosed group (n = 21)	Incorrectly diagnosed group (n = 34)
Patient characteristics		55	21 (38.2)	34 (61.8)
Demographics				
Sex	Male (%)	25 (45.5)	8 (38.1)	17 (50.0)
	Female (%)	30 (54.5)	13 (61.9)	17 (50.0)
Age	Median (year, range)	26 (0–78)	17 (2–78)	36 (0–78)
Timeline information				
Duration from awareness to initial visit (days)		183 (7–3600)	300 (28–1202)	113.5 (7–3600)
Duration from awareness to surgery (days)		213 (14–3600)	374 (47–1262)	157 (14–3600)
Clinical features				
Mobility (%)		30 (54.5)	12 (57.1)	18 (52.9)
Presence of telangiectasia (%)		8 (14.5)	4 (19.0)	4 (11.8)
Lesion size (mm, range)		10 (3–70)	12 (3–70)	10 (3–35)
Lesion type	Subcutaneous nodule (%)	41 (74.6)	20 (95.2)	21 (61.8)
	Nodule (%)	12 (21.8)	1 (4.8)	11(32.3)
	Papule (%)	2 (3.6)	0 (0.0)	2 (5.9)
Localization	Head (%)	21 (38.2)	6 (28.6)	15 (44.1)
	Neck (%)	4 (7.3)	1 (4.8)	3 (8.8)
	Trunk (%)	4 (7.3)	1 (4.8)	3 (8.8)
	Upper limb (%)	24 (43.6)	12 (57.0)	12 (35.4)
	Lower limb (%)	2 (3.6)	1 (4.8)	1 (2.9)
Color characteristics				
Color pattern	Single-colored (%)	43 (78.2)	17 (81.0)	26 (76.5)
	Multicolored (%)	12 (21.8)	4 (19.0)	8 (23.5)
Color type	Reddish (%)	23 (34.3)	5 (20.0)	18 (42.9)
	Bluish (%)	23 (34.3)	14 (56.0)	9 (21.4)
	Brownish (%)	7 (10.4)	1 (4.0)	6 (14.3)
	Yellowish-white (%)	14 (21.0)	5 (20.0)	9 (21.4)
Pathological features				
Presence of calcification (%)		22 (40.0)	10 (47.6)	12 (35.3)
Ratio of basophilic cells and shadow cells (range)		0.10 (0.00–2.19)	0.06 (0.00–0.92)	0.13 (0.00–2.19)

**Figure 1:**
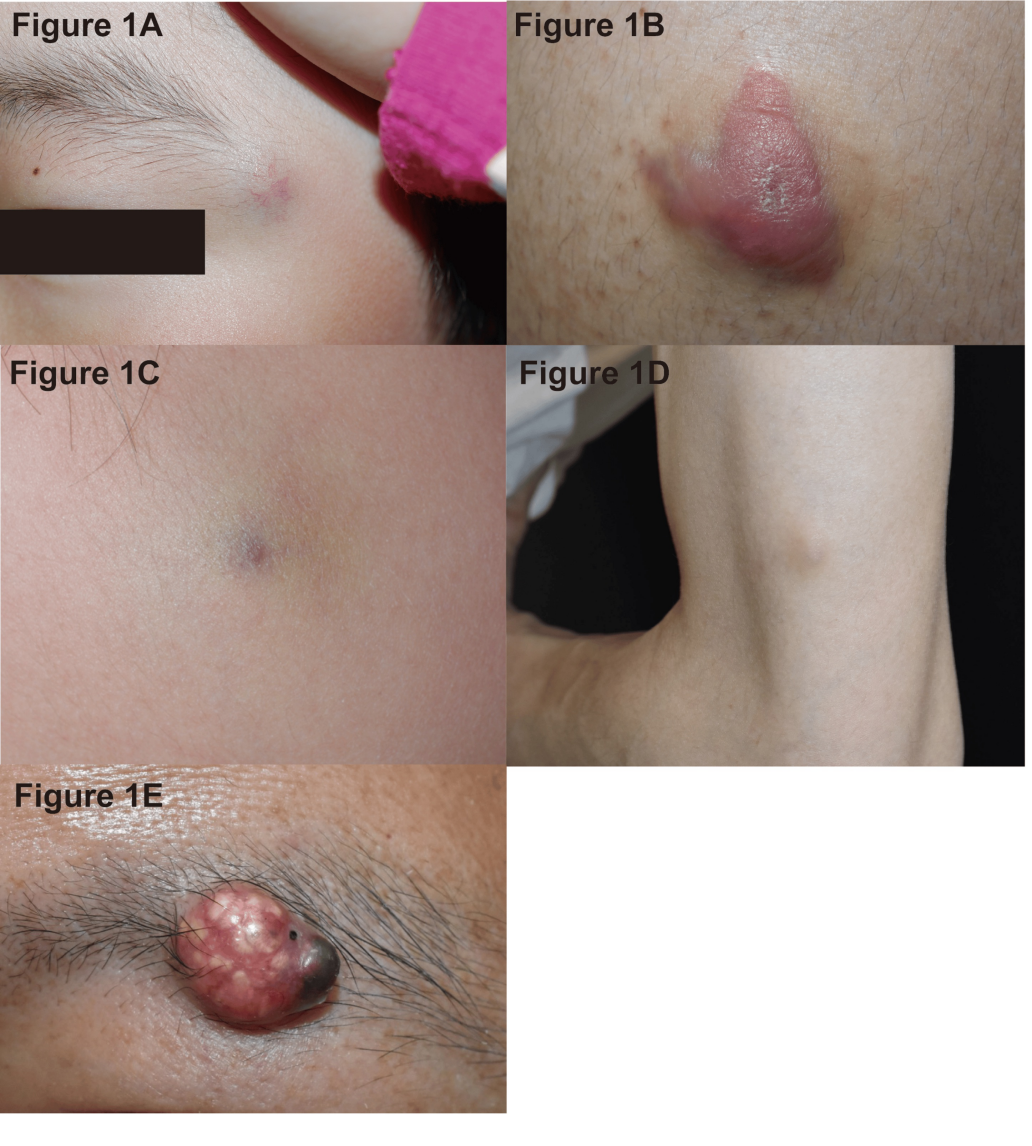
Clinical appearance of pilomatricoma in the present study. (A) A 5-year-old girl with a bluish nodule in the left lateral canthus; presented as a typical case. (B) A 21-year-old man with a reddish nodule on the left upper arm. (C) A 6-year-old boy with a bluish nodule on the right cheek. (D) A 41-year-old woman with a brownish nodule on the left upper arm. (E) A 53-year-old man with a yellowish-white nodule on the left eyebrow.

Most lesions (43 cases, 78.2%) were single-colored. Regarding color type, reddish and bluish tones were each noted in 23 of 67 observations (34.3%), followed by yellowish-white in 14 (21.0%) and brownish in 7 (10.4%). Some lesions exhibited multiple colors; thus, percentages were calculated based on the total number of color observations (n = 67). Representative clinical photographs illustrating the color diversity of pilomatricoma are shown in Figure 1.

Calcification was observed in 22 cases (40.0%), and the median B/S ratio was 0.10 (range: 0.00-2.19). Although not included in Table 1, initial clinical diagnoses at the first visit were pilomatricoma (n = 21, 38.2%), epidermal cyst (n = 24, 43.6%), dermatofibroma (n = 2, 3.6%), dermatofibrosarcoma protuberans (DFSP; n = 2, 3.6%), sebaceous neoplasm (n = 2, 3.6%), cutaneous lymphoid hyperplasia (n = 1, 1.8%), follicular neoplasm (n = 1, 1.8%), hemangioma (n = 1, 1.8%), and hypertrophic scar (n = 1, 1.8%).

Univariate analysis

Univariate analyses were performed to identify factors associated with correct initial diagnosis. Patients in the correctly diagnosed group were significantly younger than those in the incorrectly diagnosed group (median: 17 vs. 36 years, p = 0.002). The duration from awareness to initial visit was also significantly longer in the correctly diagnosed group (median: 300 vs. 113.5 days, p = 0.03). Although the median ratio of basophilic cells to shadow cells was lower in the correctly diagnosed group (0.06 vs. 0.13), this difference did not reach statistical significance (p = 0.07). No significant difference was observed in lesion size between the two groups (Table 2).

**Table 2:** Univariate analysis of continuous variables between correctly and incorrectly diagnosed groups. Values are presented as median (interquartile range). Comparisons were performed using the Mann–Whitney U test. The table includes W-values and two-sided p-values. A p-value < 0.05 was considered statistically significant. P* < 0.05; P** < 0.01. CI = confidence interval; IQR = interquartile range.

Variable	Correctly diagnosed group (Median (IQR))	Incorrectly diagnosed group (Median (IQR))	W-value	p-value	Median difference (95% CI)
Age	17 (6–28)	36 (21.5–55.3)	536.5	0.002**	18 (7–30)
Duration from awareness to initial visit	300 (127.0–360.0)	113.5 (37.3–235.5)	230.5	0.03*	–104 (–202––4)
Lesion size	12 (8–22.2)	10 (6–19.5)	288.0	0.24	–2.5 (–6.4–2.0)
The ratio of basophilic cells and shadow cells	0.06 (0.02–0.16)	0.13 (0.06–0.31)	462.0	0.07	0.05 (–0.00–0.13)

Regarding categorical variables, the presence of blue color in the lesion was significantly more frequent in the correctly diagnosed group (14/21, 66.7%) than in the incorrectly diagnosed group (9/34, 26.5%) (p = 0.005; OR = 0.19, 95% CI: 0.04-0.67). Lesions with red coloration were more frequently observed in the incorrectly diagnosed group (5/21, 23.8% vs. 18/34, 52.9%) (p = 0.04; OR = 0.28, 95% CI: 0.06-1.05), although the confidence interval included 1.0. Other factors, including sex, localization (head and neck vs. other), color pattern (single vs. multicolored), mobility, and presence of telangiectasia, were not significantly associated with diagnostic accuracy (Table 3).

**Table 3:** Univariate analysis of categorical variables associated with diagnostic accuracy. Data are presented as number (percentage). Odds ratios (OR) and 95% confidence intervals (CI) were calculated using Fisher’s exact test. P* < 0.05; P** < 0.01.

Variable	Category	Correctly diagnosed group (n=21)	Incorrectly diagnosed group (n=34)	p-value	OR (95% CI)
Sex	Male	8 (38.1)	17 (50.0)	0.41	0.62 (0.17–2.12)
	Female	13 (61.9)	17 (50.0)		
Localization	Head and neck	7 (33.3)	18 (52.9)	0.17	0.45 (0.12–1.56)
	Other	14 (66.7)	16 (47.1)		
Color pattern	Single-colored	17 (81.0)	26 (76.5)	0.75	0.77 (0.14–3.44)
	Multicolored	4 (19.0)	8 (23.5)		
Color of tumor (Blue)	Blue	14 (66.7)	9 (26.5)	0.005**	0.19 (0.04–0.67)
	Not blue	7 (33.3)	25 (73.5)		
Color of tumor (Red)	Red	5 (23.8)	18 (52.9)	0.04*	0.28 (0.06–1.05)
	Not red	16 (76.2)	16 (47.1)		
Mobility	Yes	12 (57.1)	18 (52.9)	0.78	1.18 (0.35–4.11)
	No	9 (42.9)	16 (47.1)		
Telangiectasia	Yes	4 (19.0)	4 (11.8)	0.46	1.75 (0.28–10.69)
	No	17 (81.0)	30 (88.2)		

These findings suggest that younger age, prolonged time before presentation, and the presence of bluish coloration may be associated with a correct initial diagnosis.

Multivariable logistic regression analysis

Table 4 shows the results of the multivariable logistic regression analysis for factors associated with correct initial diagnosis. Variables included in the model were selected based on the results of univariate analysis, focusing on those with statistically significant associations (p < 0.05), while taking into account the limited sample size to avoid model overfitting. None of the included variables reached statistical significance in the multivariable analysis with diagnostic accuracy, including age (OR = 0.96, 95% CI: 0.93-1.00, p = 0.07), tumor color, and the duration from symptom awareness to initial visit. The model intercept was statistically significant (OR = 3.50, 95% CI: 1.01-12.10, p = 0.04); however, this simply reflects the baseline odds of a correct diagnosis under the reference conditions and does not imply any specific clinical predictor.

**Table 4:** Multivariable logistic regression analysis of factors associated with correct initial diagnosis. Odds ratios (OR), 95% confidence intervals (CI), and p-values are shown for each variable included in the model. P* < 0.05 was considered statistically significant.

Variable	Odds ratio (95% CI)	p-value
Intercept	3.50 (1.01 – 12.10)	0.04*
Age	0.96 (0.93 – 1.00)	0.07
Tumor color (not blue)	0.34 (0.09 – 1.32)	0.12
Tumor color (reddish)	0.50 (0.12 – 1.98)	0.32
Duration from awareness to initial visit	1.00 (0.99 – 1.00)	0.75

Spearman’s rank correlation analysis

**Figure 2:**
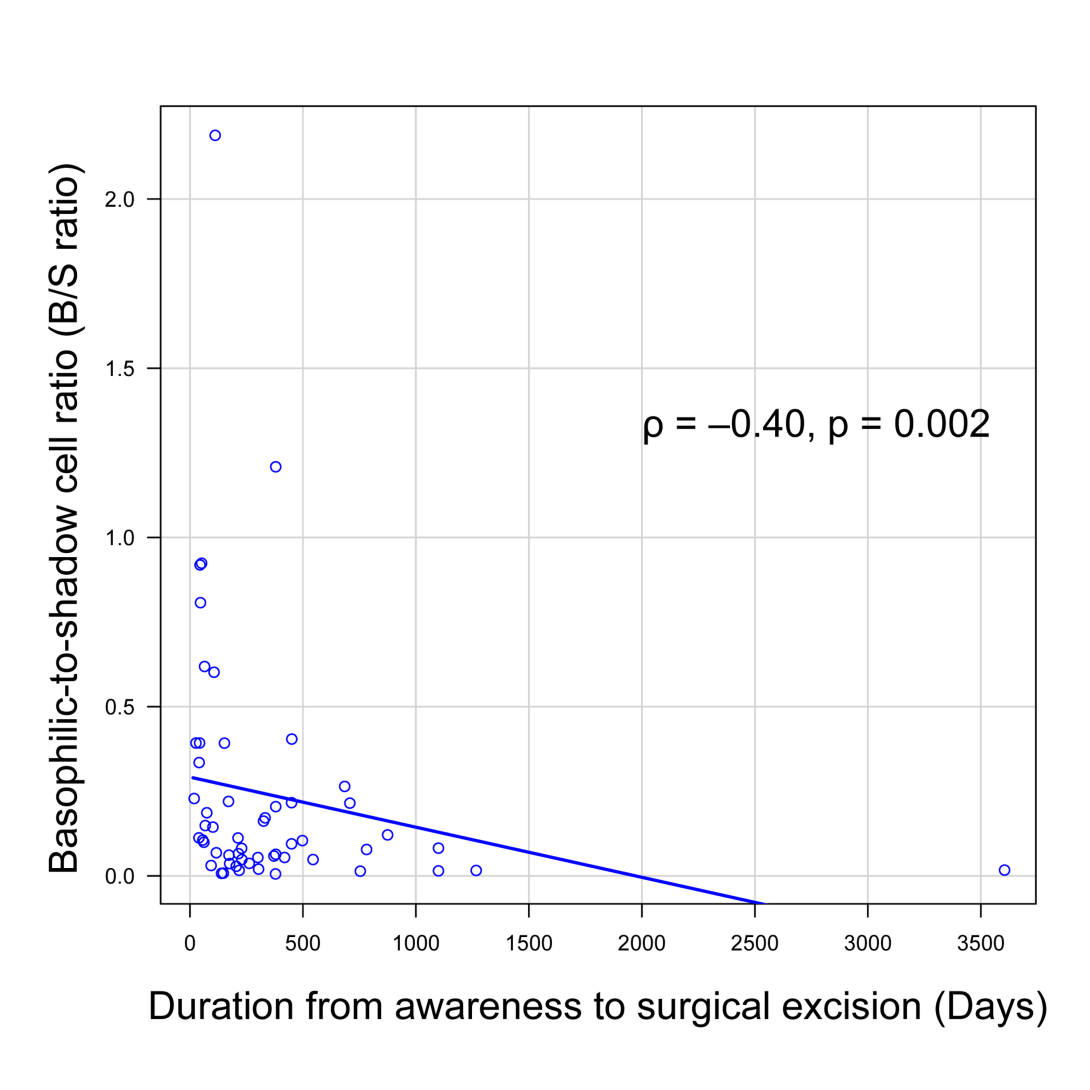
Correlation between the basophilic-to-shadow cell (B/S) ratio and duration from symptom awareness to surgical excision. A significant negative correlation was observed between the B/S ratio and the time to surgery (ρ = –0.40, p = 0.002), calculated using Spearman's rank correlation. A p-value < 0.05 was considered statistically significant.

Figure 2 shows a scatter plot illustrating the negative association between the B/S ratio and the duration from symptom onset to surgery. A significant negative correlation was observed (Spearman's rho = -0.40, p = 0.002).

## Discussion

This retrospective analysis investigated the clinical and histopathological features of pilomatricoma and the diagnostic accuracy at the time of initial presentation. Our findings highlight several key factors that may influence whether pilomatricoma is correctly diagnosed at the first clinical encounter.

The overall diagnostic accuracy in our study was 21 out of 55 cases (38.2%), consistent with previous reports indicating that the correct presurgical identification of pilomatricoma remains challenging despite its benign nature and relatively high prevalence in pediatric populations [11,12]. The low accuracy observed may reflect the variable clinical appearance of pilomatricoma, which can mimic other benign or malignant cutaneous lesions [14]. Furthermore, given that our institution is a university hospital, a referral bias likely exists, with more diagnostically difficult or atypical cases being sent for evaluation. Several cases were referred specifically for excision at the request of the patient or referring physician, possibly skewing the cohort toward diagnostically ambiguous presentations.

Age was significantly associated with correct diagnosis in the univariate analysis. This aligns with the known epidemiology of pilomatricoma, which predominantly affects children and adolescents [4,5]. Clinicians may be more likely to consider pilomatricoma in younger patients when evaluating firm cutaneous nodules. Conversely, lesions in older adults may prompt consideration of alternative diagnoses, leading to a lower diagnostic yield in that population.

Lesion color also appeared to influence diagnostic accuracy. The presence of bluish tones was significantly associated with correct diagnosis, while reddish lesions were more frequently observed in the incorrectly diagnosed group, although this association should be interpreted with caution due to the confidence interval including 1.0. These findings suggest that clinicians may rely on classic descriptions of pilomatricoma, such as bluish or skin-colored firm nodules when forming an initial impression. Importantly, the presence of multiple colors within a lesion did not significantly affect diagnostic accuracy, highlighting that clinicians may prioritize specific tones such as blue, over overall color complexity.

Interestingly, lesions located on the upper limbs were most common in our cohort (24 cases, 43.6%), followed by the head and neck. Although pilomatricoma has traditionally been described as most frequently occurring in the head and neck region, recent studies in Japanese populations have noted a higher proportion of upper limb lesions [15]. Our findings support this regional trend and emphasize the importance of considering demographic and ethnic variations in clinical presentation. All patients in our study were Japanese, further underscoring the relevance of regional and ethnic factors in clinical characteristics and diagnostic approaches.

The interval between symptom awareness and the initial visit was significantly longer in the correctly diagnosed group. This may reflect a temporal maturation of the lesion, with more time allowing for calcification and other characteristic features to develop, making diagnosis more straightforward. In contrast, rapidly growing or recently noticed nodules may raise concerns for malignancy, diverting the clinical impression away from pilomatricoma.

Histologically, we observed a moderate negative correlation between the B/S ratio and the duration from symptom onset to surgery. This aligns with the known histologic progression of pilomatricoma, in which basophilic cells gradually transition into anucleate shadow cells over time [16,17]. Although the exact timing of lesion onset is difficult to determine, our use of patient-reported awareness provides a practical, if imperfect, proxy for early lesion development.

In the multivariable analysis, none of the examined variables reached statistical significance, which may be attributable to the limited sample size. Although all variables included in the model were selected based on significant associations in univariate analysis, these associations did not persist after adjustment for other factors. The model’s intercept was statistically significant, but this likely reflects the baseline probability of correct diagnosis under reference conditions, rather than the presence of a specific clinical predictor. These findings underscore the complexity of clinical diagnosis and highlight the importance of routinely considering pilomatricoma in the differential diagnosis of cutaneous nodules, regardless of patient age or other clinical features.

This study has several limitations, including its retrospective design and relatively small sample size. The reliance on clinical documentation for color description and lesion characteristics may have introduced observer bias. Furthermore, the date of symptom awareness was patient-reported and subject to recall bias.

## Conclusions

In conclusion, our findings reaffirm the diagnostic challenges associated with pilomatricoma and suggest that younger age, prolonged lesion duration, and bluish coloration may aid in clinical recognition. Further prospective studies with larger cohorts and standardized diagnostic criteria are warranted to enhance early identification of this common but frequently misdiagnosed tumor.
